# A Mendelian randomization analysis of cardiac MRI measurements as surrogate outcomes for heart failure and atrial fibrillation

**DOI:** 10.1038/s43856-025-00855-1

**Published:** 2025-04-19

**Authors:** A. F. Schmidt, C. Finan, J. van Setten, E. Puyol-Antón, B. Ruijsink, M. Bourfiss, A. I. Alasiri, B. K. Velthuis, F. W. Asselbergs, A. S. J. M. te Riele

**Affiliations:** 1 Institute of Cardiovascular Science, Faculty of Population Health, University College London, London, UK; 2 UCL BHF Research Accelerator Centre, London, UK; 3 Department of Cardiology, Division Heart and Lungs, University Medical Center Utrecht, Utrecht University, Utrecht, The Netherlands; 4 Department of Cardiology, Amsterdam Cardiovascular Sciences, Amsterdam University Medical Centre, University of Amsterdam, Amsterdam, The Netherlands; 5 Department of Biomedical Engineering, School of Biomedical Engineering and Imaging Sciences, King’s College London, King’s Health Partners, London, UK; 6 School of Imaging Sciences and Biomedical Engineering, King’s College London, London, UK; 7 Medical Genomics Research Department, King Abdullah International Medical Research Center, King Saud Bin Abdulaziz University for Health Sciences, Ministry of National Guard Health Affairs, Riyadh, Saudi Arabia; 8 Department of Radiology, University Medical Center Utrecht, Utrecht University, Utrecht, The Netherlands; 9 Institute of Health Informatics, Faculty of Population Health, University College London, London, UK; 10 Netherlands Heart Institute, Utrecht, the Netherlands

**Keywords:** Cardiology, Arrhythmias, Heart failure

## Abstract

**Background:**

Drug development and disease prevention of heart failure (HF) and atrial fibrillation (AF) are impeded by a lack of robust early-stage surrogates. We determined to what extent cardiac magnetic resonance (CMR) measurements act as surrogates for the development of HF or AF.

**Methods:**

Genetic data were sourced on the association with 21 atrial and ventricular CMR measurements. Mendelian randomization was used to determine CMR associations with AF, HF, non-ischaemic cardiomyopathy (NICM), and dilated cardiomyopathy (DCM), noting that the definition of NICM includes DCM as a subset. Additionally, for the CMR surrogates of AF and HF, we explored their association with non-cardiac traits potentially influenced by cardiac disease liability.

**Results:**

In total we find that 7 CMR measures (biventricular ejection fraction (EF) and end-systolic volume (ESV), as well as LV systolic volume (SV), end-diastolic volume (EDV), and mass to volume ratio (MVR)) associate with the development of HF, 5 with the development of NICM (biventricular EDV and ESV, LV-EF), 7 with DCM (biventricular EF, ESV, EDV, and LV end-diastolic mass (EDM), and 3 associate with AF (LV-ESV, RV-EF, RV-ESV). Higher EF of both ventricles associate with lower risk of HF and DCM, with biventricular ESV associating with all four cardiac outcomes. Higher values of biventricular EDV associate with lower risk of HF, and DCM. Exploring the associations of these CMR cardiac disease surrogates with non-cardiac traits confirms a strong link with diastolic blood pressure, as well as more specific associations with lung function (LV-ESV), HbA1c (LV-EDM), and type 2 diabetes (LV-SV).

**Conclusions:**

The current paper identifies key CMR measurements that may act as surrogate endpoints for the development of HF (including NICM and DCM) or AF.

## Plain language summary

Heart failure and irregular heart rhythms are common and serious heart conditions. Finding early warning signs could help prevent these diseases or enable the development of better treatments. This study looked at heart measurements taken using heart scans to see whether these could act as early indicators of future heart problems. Using information about the genes people had inherited, we investigated whether changes in heart size or function are linked to a person’s risk of developing heart disease. We found several measurements that are strongly linked to later heart failure or irregular heart rhythms. These findings suggest that heart-based measurements could help identify people at higher risk of heart problems earlier. In the future, this might help guide treatment decisions or identify new ways to prevent heart disease.

## Abstract (web summary)

Schmidt et al. use Mendelian randomization to identify (biventricular) cardiac MRI measurements with a strong association with heart failure and/or atrial fibrillation. Nine cardiac MRI measurements are identified that may be relevant to consider as surrogate outcomes in both research and clinical settings.

## Introduction

Heart failure (HF) and atrial fibrillation (AF) are major cardiac diseases that cause a considerable burden in terms of health and economic costs, as well as mortality^1–3^. HF is a clinical diagnosis secondary to dysfunction of the right ventricle (RV) or left ventricle (LV), while AF is defined by uncoordinated electrical activation and consequently ineffective contraction of the atria. Both diseases are intricately related, and while the causative relationship between the two conditions has not been fully determined, it is clear these two diseases frequently co-occur^4^.

Despite recent advances in medicines, for example, offered by sodium-glucose co-transporter-2 inhibitors, drug development for cardiac disease suffers from high failure rates, often occurring during costly late-stage clinical testing^5–7^. Unlike the cholesterol content on low-density lipoprotein particles for coronary heart disease, drug development for AF and HF is impeded by a lack of robust early-stage surrogates (or intermediates) for cardiac disease.

Cardiac magnetic resonance (CMR) imaging is the gold standard for the quantification of atrial and ventricular function and morphology and has become an integral diagnostic modality for cardiac diseases. It is, however, unclear to what extent CMR measurements act as surrogates for the development of cardiac disease in otherwise healthy individuals.

Both HF and AF are associated with multimorbidity, including non-cardiac diseases, such as stroke, chronic kidney disease (CKD), diabetes mellitus (T2DM), and neurological diseases, such as Alzheimer’s disease (AD). Because HF and AF are clinical manifestations of underlying changes in cardiac function and structure, patients with similar diagnoses may vary considerably in underlying pathophysiology and disease progression. Unlike HF or AF diagnoses, CMR measurements directly reflect cardiac physiology and therefore, provide an opportunity to explore the effects changes in cardiac function and structure may elicit in other organs.

Recently, CMR measurements of thousands of subjects have been linked to genetic data and analysed through genome-wide association studies (GWAS). Aggregate data from GWAS, consisting of variant-specific point estimates and standard errors, can be used in Mendelian randomization analyses to ascertain the causal effects a CMR trait may have on disease. In the current manuscript, we leveraged data from two recent GWAS of CMR measurements of cardiac structure and function^8^, and left atrial (LA) volume^9^, jointly consisting of 21 measurements conducted in over 35,000 UK biobank (UKB) participants. These data were used to conduct Mendelian randomization analyses to determine the potential association between CMR traits and cardiac events, specifically focussing on AF and HF, as well as the aetiological HF subtypes: dilated cardiomyopathy (DCM) and non-ischaemic cardiomyopathy (NICM)^10,11^. Subsequently, we explored the association of CMR surrogates for HF or AF with 19 clinically relevant non-cardiac traits, focussing on traits potentially influenced by cardiac disease liability, such as blood pressure, kidney and lung function markers, diabetes, and stroke subtypes. In this study, we identify seven CMR measures associated with the development of HF, seven with DCM, five with the development of NICM, and three with AF. Moreover, we find that many of these CMR measures are associated with diastolic blood pressure, while specific individual measures also relate to lung function and glucose metabolism.

## Methods

### Genetic data on CMR and cardiac traits

We leveraged aggregate data (i.e. point estimates and standard errors) from two GWAS of deep-learning derived CMR measurements conducted using UKB participants; please see the Supplementary Methods and the specific study references for details on the derivation methods. Ahlberg et al.^9^ provided measurements on LA volume (LA-V (max) and LA-V (min)), LA total emptying fraction (LA-TF), LA active emptying fraction (LA-AF), and passive emptying fraction (LA-PF) from 35,658 subjects. Schmidt et al.^8^ provided (n: 36,548) data on biventricular ejection fraction (EF), stroke volume (SV), peak filling rate (PFR), peak atrial filling rate (PAFR) peak ejection rate (PER), end-diastolic or end-systolic volumes (EDV, ESV), LV end-diastolic mass (LV-EDM), and LV mass to volume ratio (LV-MVR). The deep-learning algorithms employed for automated CMR analyses included outlier detection steps and removing CMRs from the minority of subjects who were, for example, out of sinus rhythm.

GWAS data were included on the following cardiac outcomes: HF (52,496 cases)^12^, NICM (1816 cases)^13^, DCM (2719 cases)^14^, and AF (60,620 cases)^15^. Here, NICM and DCM were sourced from distinct samples, where the phenotypic overlap between DCM and NICM (with DCM being a more homogenous subgroup of NICM) allowed for indirectly replication of our findings. The following 19 traits were used in the non-cardiac phenome-wide scan: five stroke subtypes^16^, venous thromboembolism (VTE)^17^, abdominal aortic aneurysm (AAA)^17^, systolic/diastolic blood pressure (SBP/DBP)^18^, body mass index (BMI)^19^, T2DM^20^, glycated haemoglobin (HbA1c) from the Neale UKB analysis (http://www.nealelab.is/uk-biobank), C-reactive protein (CRP)^21^, lung function measurement from the Neale UKB analysis (http://www.nealelab.is/uk-biobank, forced expiratory volume: FEV1, forced vital capacity: FVC, peak expiratory flow: PEF), CKD^22^, estimated glomerular filtration rate (eGFR)^22^, and AD^23^; please see the data availability section for more detail. The original GWA studies sought to prevent bias due to population stratification or cryptic relatedness by either removing non-European individuals (based on genetic principal component analysis), related individuals, or accounting for this through mixed-effects models such as BOLT-LMM.

### Mendelian randomization analysis

Genetic instruments were selected from throughout the genome using an F-statistic > 24 and a minor allele frequency of at least 0.01. Variants were clumped to a linkage disequilibrium (LD) R-squared threshold of 0.30, with residual LD modelled using a generalized least square (GLS) solution^24^ and a reference panel from a random sample of 5000 of white British ancestry UKB participants. By actively modelling the remaining LD structure, the employed GLS estimators prevent potential bias in the standard error estimates due to the correlation between variants while at the same time optimising precision and stability by including additional variants^24^. To maximise the number of available variants, we did not perform any additional distance-based clumping. Steiger filtering^25^ was employed to remove variants with a likely direct causal effect on the outcome instead of on the exposure (i.e., removing variants affecting the outcome before the change in exposure occurred: variant -> outcome -> exposure), ensuring the remaining genetic instruments supported an association model where the exposure occurred before the outcome.

Mendelian randomization was conducted using the GLS implementation of the inverse-variance weighted (IVW) estimator, as well as with an Egger correction to protect against horizontal pleiotropy^26^. To further minimize the potential influence of horizontal pleiotropy, we excluded variants with a leverage value of more than 3 times the mean or an outlier Chi-square statistic above 11, with the Q-statistic identifying possible remaining violations^27^. Noting the lack of power of the Egger intercept test, we instead report the *p*-value of the Q-test^27^. The Rucker model selection framework was applied to select the most appropriate estimator (IVW or MR-Egger) for each individual exposure-outcome relation^27,28^. To ensure a sufficient number of variants were available to accurately explore possible horizontal pleiotropy effects, we dropped CMR measurement with less than 5 variants. The influence of the horizontal pleiotropy assumption was additionally evaluated using the weighted median estimator, which assumes at least 50% of the information is derived from valid instruments. Importantly, other than assuming at least 50% of the variants are valid instruments, the median estimator does not make any specific assumptions on the type of horizontal pleiotropy affecting the invalid genetic instruments. The weighted median estimator, therefore, provides a middle ground between the IVW estimator (which assumes the complete absence of horizontal pleiotropy) and the MR-Egger estimator (which allows for 100% of the variants acting through a horizontal pleiotropy pathway)^29,30^. Given that the Median estimator does not allow for the inclusion of correlated variants, the genetic instruments used in this analysis were pruned to an R-squared of 0.05.

Previous Mendelian randomisation studies have often applied a *p*-value threshold of 5 × 10^-8^ (approximately equal to an F-statistic of 30) to identify instruments with a sufficiently strong exposure association. While this conservative threshold does protect against weak-instrument bias, applying a lower F-statistic threshold may beneficially increase the number of available variants and thereby decrease the type 2 error rate. We ensured the presented results remained sufficiently protected against the potential influence of weak-instrument bias by applying a reasonably high F-statistic threshold and by prioritising outcome GWAS with limited overlap with UKB data used by the CMR studies^31^. Nevertheless, the following outcomes were based on GWAS where over 50% of the participants were sourced from the UKB: AF, NICM, SBP & DBP, BMI, HbA1c, lung function metrics. It is therefore important to note that in large sample size settings (where the estimated F-statistic converges to the true F-statistic) the multiplicative inverse of the estimated F-statistic approximates the amount of bias in one-sample settings (with complete sample overlap), in our cases this implies the average amount of potential bias with complete sample overlap is about 4%^31^.

Where appropriate, results were presented as odds ratio (OR, for binary traits) with 95% confidence interval (95%CI) or mean difference (MD, for continuous traits). Given the close interrelationship between CMR traits themselves, as well as between the considered cardiac traits (e.g., with HF, DCM, and NCIM reflecting closely related diseases) we sought to identify the subset of CMR traits with strong support for cardiac involvement. Importantly, because of the aforementioned phenotypic interrelationship, associations with multiple cardiac traits should be viewed as supportive rather than penalised. We, therefore, applied a two-stage approach to address multiple testing. First, individual associations with cardiac outcomes were declared significant using the standard alpha of 0.05. Next, following Storey^32^, multiplicity was addressed by using Kolmogorov-Smirnov “KS”-tests to de-prioritise CMR traits for which the *p*-value distribution followed a uniform distribution indicative of an overall null-association. Focussing on the CMR traits that were rejected by the KS-tests we conducted a targeted phenome-wide scan to explore their potential association with the aforementioned non-cardiac traits, applying a multiplicity corrected alpha of 2.63 × 10^-4^ (correcting for the 10 remaining CMR traits and 19 exposure).

As outlined in the supplementary methods, we additionally investigate the extent to which increased HF liability is associated with a rise in drug prescriptions^33^, which might act as mediators.

### Institutional review board approval

Aside from the UKB LD reference data, the current study exclusively uses summary-level GWAS statistics, with download URLs provided in the data availability section. For all included GWA studies, all participants provided informed consent, and study protocols were approved by their respective local ethical committee. The UK Biobank has ethical approval from the North West Multi-centre Research Ethics Committee to handle human participant data. Written informed consent was obtained from all participants, and all data was deidentified for analysis. This research has been conducted using the UK Biobank Resource under Application Number 12113. Given that the current study uses aggregate GWAS data, we did not seek further ethical approval.

### Reporting summary

Further information on research design is available in the Nature Portfolio Reporting Summary linked to this article.

## Results

### Biventricular and atrial CMR associations with cardiac outcomes

We employed Mendelian randomization to determine the potential association between CMR traits and the liability of AF, HF, DCM, and/or NICM. Due to the limited number of available variants (fewer than 5), the following CMR parameters were excluded from the analysis: LA-PF and LA-TF, biventricular PAFR and PFR, as well as LV-PER.

**Fig. 1:**
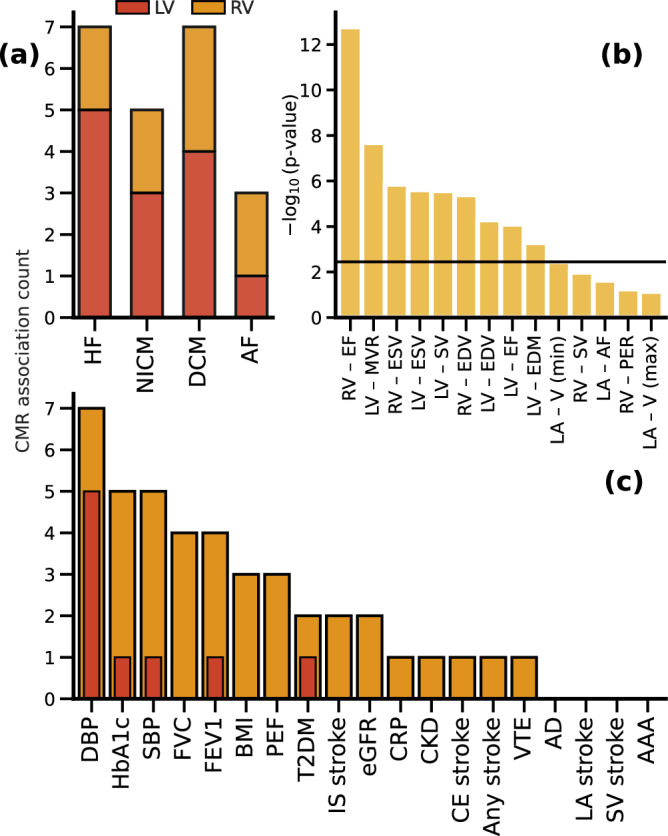
Kolmogorov-Smirnov test results, and CMR association counts with cardiac and non-cardiac traits. Nota bene: the bar chart in (**a**) represent the -log_10_ (*p*-value) of Kolmogorov-Smirnov test results, were a significant *p*-value indicates the associations are unlikely driven by multiple testing. The horizontal line indicates the significance threshold of 0.05/14 – representing the 14 CMR traits with at least five available variants in the cardiac outcome analysis. The bar chart (**b**) represents the counts of significant Mendelian randomization CMR effects grouped by chamber. These counts disregard CMRs which did not pass the Kolmogorov-Smirnov test for multiplicity, and are based on the weighted median results. The bar chart in (**c**) represents the counts of significant Mendelian randomization CMR effects on the considered phenome-wide traits. Results are based on the CMRs which passed Kolmogrov-Smirnov test for multiplicity, with the Rucker selected (IVW/Egger) results depicted as the wider yellow bars, and the thinner red bars representing the weighted median results. The following abbreviations were used, LV left-ventricle, RV right-ventricle, RA right-atrial, LA right-atrial, HCM hypertrophic cardiomyopathy, DCM dilated cardiomyopathy, AF atrial fibrillation, T2DM type 2 diabetes, CKD chronic kidney disease, VTE venous thromboembolism, AAA abdominal aortic aneurysm, SBP/DBP systolic/diastolic blood pressure, BMI body mass index, CRP c-reactive protein, FVC forced vital capacity, FEV1 forced expiratory volume, PEF peak expiratory flow, eGFR: estimated glomerular filtration rate, HbA1c glycated haemoglobin, CE cardioembolic, LA large artery, IS ischaemic, SV small vessel, AD Alzheimer’s disease. See Data 1–4 for the underlying data.

**Fig. 2:**
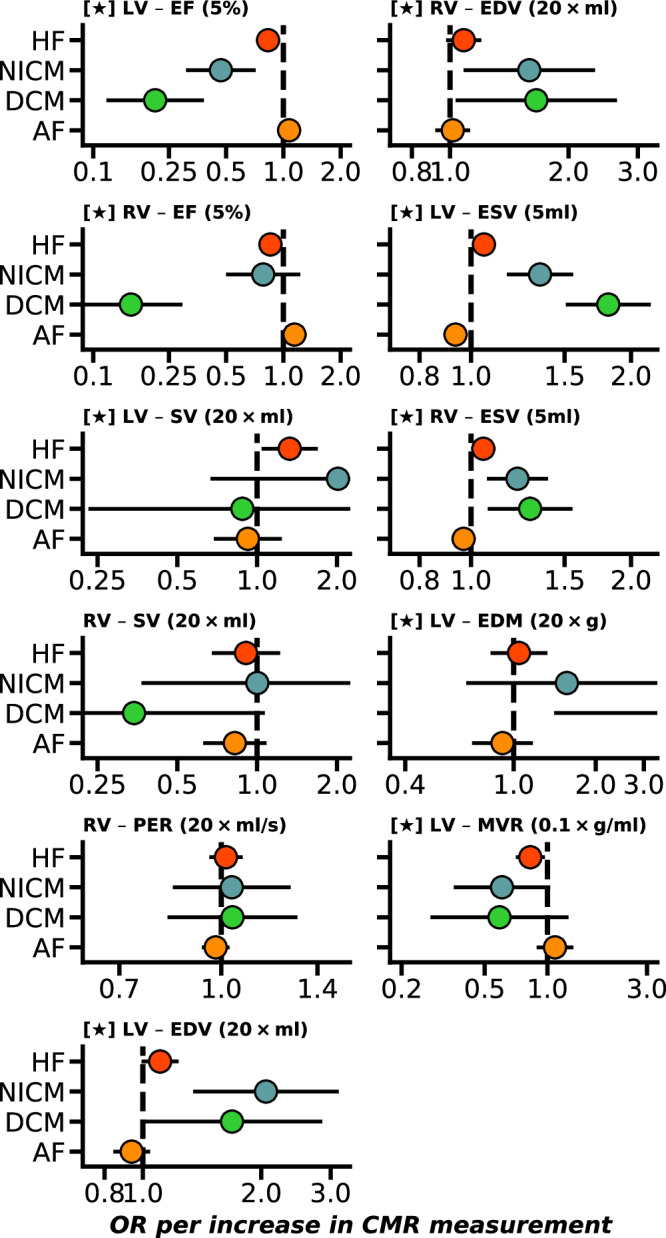
Weighted median Mendelian randomization estimates of biventricular CMR associations with the onset of heart failure and atrial fibrillation. Nota bene: point estimates reflect odds ratios (OR) with 95% confidence intervals presented as horizontal line segments. CMR measurements that passed the Kolmogorov-Smirnov test for multiplicity are indicated with a star. LV left-ventricle, RV right-ventricle, EF ejection fraction, SV stroke volume, PFR peak filling rate, PER peak ejection rate, EDV/ESV diastolic or systolic volumes, EDM end diastolic mass, MVR mass to volume ratio. Outcome data were available on HF (heart failure, 52,496 cases), DCM (dilated cardiomyopathy, 2719 cases), NICM (non-ischaemic cardiomyopathy, 1816 cases), and AF (atrial fibrillation, 60,620 cases). See Data 1 and 2 for the underlying data.

**Fig. 3:**
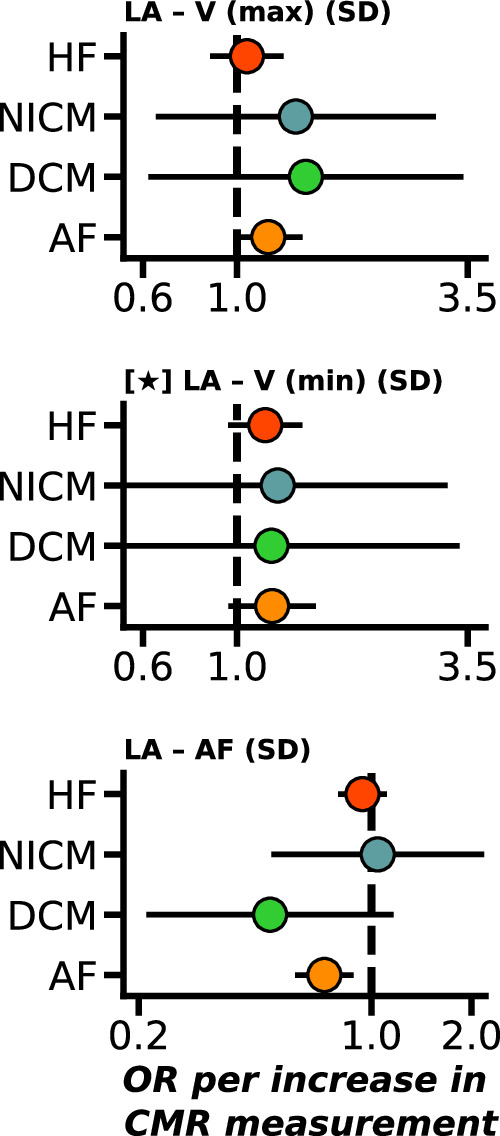
Weighted median Mendelian randomization estimates of atrial CMR associations with the onset of ﻿heart failure and atrial fibrillation. Nota bene: point estimates reflect odds ratios (OR) with 95% confidence intervals presented as horizontal line segments. CMR measurements that passed the Kolmogorov-Smirnov test for multiplicity are indicated with a star. LA left-atrial, V (max) maximum volume, V (min) minimum volume, AF active emptying fraction, PF passive emptying fraction, PFR peak filling rate. Outcome data were available on HF (heart failure, 52,496 cases), DCM (dilated cardiomyopathy, 2719 cases), NICM (non-ischaemic cardiomyopathy, 1816 cases), and AF (atrial fibrillation, 60,620 cases). See Data 2 for the underlying data. See Data 1 and 2 for the underlying data.

Prioritising the initial Rucker based MR analysis (Supplementary Figs. 1, 2, Data 1) on results which passed multiplicity filtering (Fig. 1), we identified 10 CMR traits which were associated (multiple) cardiac outcomes: biventricular EF, ESV, EDV, LV-SV, LV-EDM, LV-MVR, LA -V (min). We subsequently compared these Rucker-based point estimates to those obtained using the weighted median estimator (which assumes horizontal pleiotropy is absent in at least 50% of the used genetic variants), which showed strong agreement: Spearman’s correlation of 0.86 (*p*-value 1.10 × 10^-11^); Figs. 2, 3, Data 2.

Focussing on the subset of estimates with a constant association across the considered MR estimators, we found that increased biventricular EF associated with lower risk of HF and DCM, with LV-EF additionally associated with lower risk of NICM. Similar biventricular associations were observed for EDV (lower risk of NICM, DCM), and ESV (higher risk of AF, HF, DCM, NICM). Additionally, higher LV-EDM was associated with higher DCM risk (OR 3.93, 95%CI 1.43; 10.83), and higher LV-SV was associated with higher HF risk (OR 1.33, 95%CI 1.06; 1.67). Contrary to the Rucker MR analysis, in the MR-median analysis, LA-V (min) did not associate with cardiac disease; Figs. 1–3.

### Associations of CMR-derived indices of cardiac function and structure with non-cardiac traits

**Fig. 4:**
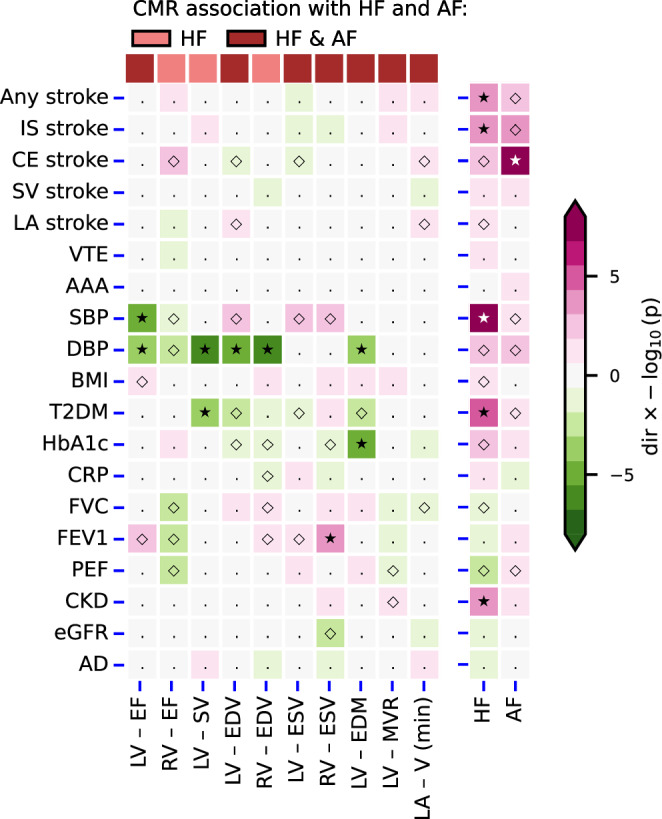
A targeted phenome-wide scan comparing the effect of changes in cardiac function and structure to those of a heart failure or atrial fibrillation diagnosis. Nota bene: *p*-values passing the 0.05 threshold are indicated by an open diamond, with stars indicating results passing a threshold of 2.6 × 10^−4^. Cells were coloured by effect direction times -log_10_(*p*-value); where *p*-values were truncated at 8 for display purposes. CMR traits were selected based on the Kolmogorov-Smirnov test for multiplicity, with effects estimated using the weighted median estimator. The following abbreviations were used, LV left-ventricle, RV right-ventricle, LA right-atrial, EF ejection fraction, SV stroke volume, EDV/ESV diastolic or systolic volumes, EDM end diastolic mass, MVR mass to volume ratio V (max): maximum volume, AF atrial fibrillation, T2DM type 2 diabetes, CKD chronic kidney disease, VTE venous thromboembolism, AAA abdominal aortic aneurysm, SBP/DBP systolic/diastolic blood pressure, BMI: body mass index, CRP c-reactive protein, FVC forced vital capacity, FEV1 forced expiratory volume, PEF peak expiratory flow, eGFR estimated glomerular filtration rate, HbA1c glycated haemoglobin, CE cardioembolic, LA large artery, IS: ischaemic, SV small vessel, AD Alzheimer’s disease. See Data 3–6 for the underlying data.

Focusing on the same 10 CMR measurements, we next explored whether changes in cardiac function and structure could be associated with non-cardiac traits. We note that the correlation between the Rucker-based point estimates and weighted median-based estimates was lower for non-cardiac trait associations: 0.54 (*p*-value 2.06 × 10^-14^), suggesting the specific horizontal pleiotropy assumption was more influential. In line with this the weighted median-based estimator identified substantially fewer significant associations with non-cardiac traits (Figs. 1 and 4, Data 3, 4, Supplementary Fig. 3, 4). Focussing on the subset of non-cardiac traits with concordant results between estimators identified five CMR traits which were strongly associated with a decrease in DPB (LV-EF, LV-SV, LV-EDV, RV-EDV, and LV-EDM), as well as single CMR traits associating with FEV1, HbA1c, and T2DM.

### Comparison to HF and AF associations with non-cardiac traits

Next, as comparison, we leveraged genetic instruments associated with a clinical diagnosis of HF or AF and performed Mendelian randomization to determine the association an increased liability of HF or AF had on non-cardiac traits (Fig. 4, Supplementary Fig. 4, Data 5, 6). An increased HF liability was strongly associated with the development of (any/ischaemic) stroke, as well as an increased blood pressure, increased risk of CKD, and T2DM. AF liability was strongly associated with the development of cardioembolic stroke. Please refer to the supplementary methods and Supplementary Table 1 for additional analyses exploring potential associations between HF liability and  drug prescription.

## Discussion

In the current manuscript, we employed Mendelian randomization combined with CMR measurements to identify surrogate outcomes for the onset of HF (52,496 cases) and AF (60,620 cases). We showed that biventricular EF was associated with the development of HF and DCM, with RV-EF additionally associating with AF. Biventricular ESV was associated with the development of all four cardiac outcomes, with biventricular EDV associating with DCM and NICM, and LV-EDV additionally with HF, and LV-EDM associating with the onset of DCM. Importantly, we observed strong consistency in terms of effect direction and magnitude across HF, DCM and NICM, which represent strongly related cardiac events. We found that the development of HF or AF reflects a combination of changes in cardiac function and structure.

In total, we identified 7 CMR measures (biventricular EF and ESV, LV-SV, LV-EDV, LV-MVR) associated with the development of HF, 5 with development of NICM (biventricular EDV and ESV, LV-EF), 7 with DCM (biventricular EF, ESV, EDV, LV-EDM), and 3 with AF (LV-ESV, RV-EF, RV-ESV). This implies that CMR measurements may be useful to monitor disease occurrence in subjects without pre-existing cardiac disease and help identify high-risk patients in need of preventative measures. Additionally, our findings suggest that CMR measurement might be used as surrogate endpoints in early clinical studies, which can assist in prioritizing compounds for confirmatory outcome trials.

We explored the phenotypic effects that changes in cardiac function and structure may have on non-cardiac traits (Figs. 1 and 4), finding strong support for an association with diastolic blood pressure. Despite a moderate correlation between the Rucker-based MR results and the weighted median results (correlation of 0.54), there was substantial disagreement between the amount of significant findings. whereas the Rucker-based method supported more prolific associations between CMR measurements and a substantial number of non-cardiac traits. The disagreement in terms of significant findings suggests that the CMR associations with non-cardiac traits are at least partially influenced by horizontal pleiotropy. The moderate correlation between point estimates does however, suggest that some of the difference in significance might be due to the lower power of the median estimator^29^. By discounting discordant results, we identified the subset of effect estimates that are relatively robust to the specific horizontal pleiotropy assumption and are, therefore, less likely to be affected by this type of bias.

We compared the CMR associations with non-cardiac traits to the associations of HF/AF liability with these same non-cardiac traits, suggesting that the associations of CMR traits and HF/AF liability are distinct. For example, a higher liability of HF was associated with a higher risk of (ischaemic) stroke, diabetes, and kidney disease, while a higher AF liability was associated with a higher cardioembolic stroke risk. While diabetes is a known risk factor for HF, we found that an increased HF liability was associated with an increased risk of diabetes. Rather than reflecting a direct effect of HF, we hypothesize that this association may reflect a mediation pathway where an increased HF liability is associated with an increased prescription rate of diabetes associated medicines^34,35^. To show this, we conducted an MR analysis of HF liability and its association with drug prescriptions using a GWAS from Wu et al.^33^, confirming that an increased risk of HF is associated with cardiovascular drug prescriptions affecting diabetes risk such as statins/HMGCR inhibitors; Supplementary Table 1.

The study has a number of limitations that deserve consideration. First, while we sourced genetic associations with CMR measurements taken from subjects without pre-existing cardiac conditions, a proportion of subjects may have had undiagnosed diseases. The UKB, however, represents a relatively healthy subset of the UK population, likely minimizing the number of individuals with latent disease. The potential influence of this is further limited by employing a two-sample MR design, where the exposure and outcome GWAS are sourced from (partially) non-overlapping samples with distinct disease patterns. This two-sample design further ensures that any potential weak-instrument bias acts towards a null effect. We do emphasise that a subset of GWAS used as a source of outcome data did include UKB data (Supplemental Table 2), which may have slightly increased anticipated bias proportional to the sample overlap (or overlap in cases for binary outcomes). For example^31^, with 100% sample overlap, our employed F-statistic (used to select instruments) would result in a small amount of bias 1/24 ≈ 0.04, with a sample overlap of 30% this would be 0.04 × 0.3 ≈ 0.01, and zero in the absence of sample overlap. As such, by combining large sample size GWAS data with limited sample overlap and relatively strong instruments the presented results are protected against potential weak-instrument bias. Second, our choice of CMR measurement was limited by the publicly available data, for example preventing us from exploring the association between the ratio of measure (such as PEF/EDV or PFR/EDV) not available in the original GWAS. Third, while Mendelian randomization is robust against bias due to reverse causality and confounding, it critically assumes the absence of horizontal pleiotropy, where the genetic variant only affects the outcome through its association with the CMR measurement. In the current analysis, we performed automatic model selection to decide between an IVW or more robust MR-egger models, removed potentially pleiotropic variants through the identification and removal of outliers and high-leverage points, and prioritised results with a significant and directionally consistent effect using the Median MR estimator. Fourth, the conducted Mendelian randomization analyses implicitly assess a linear trend between CMR and outcome. In the presence of nonlinearity, the presented Mendelian randomization estimates represent a population average effect, which may not necessarily apply to any single individual but often offers a reasonable approximation. While non-linear Mendelian randomization methods have been developed^36,37^, these require access to individual participant data, which, even for UKB-sized data, only offers a fraction of the disease cases we have been able to leverage here. Finally, due to the cumulative nature of GWAS, where results from previous studies are typically meta-analysed in subsequent studies, there was insufficient independent data to replicate our findings. Potentially, the growing number of non-European GWAS will provide avenues for cross-ancestry replication.

In conclusion, we have identified biventricular CMR measurements that may act as surrogate endpoints for future cardiac events, including heart failure, cardiomyopathies, and atrial fibrillation. We additionally show that changes in cardiac function and structure affect blood pressure, as well as identifying potential associations with lung function and glucose homoeostasis.

## Supplementary information

Supplementary Information

Description of Additional Supplementary Files

Data 1

Data 2

Data 3

Data 4

Data 5

Data 6

Data 7

REPORTING SUMMARY

## Data Availability

The source data for Fig. 1 is available as Data 1-4, the source data for Figs. 2, 3 is available as Data 1-2, the source data for Fig. 4 is available as Data 2-6. The individual variants and their trait associations have been included as Supplementary Data 7. The UKB data can be requested from www.ukbiobank.ac.uk, conditional on an approved project. Most of the GWAS data are publicly available from the following download links: for the CMR associations from Ahlberg et al. (https://zenodo.org/records/5074929) and Schmidt et al. (https://www.ebi.ac.uk/gwas/publications/37126556), for HF (http://results.globalbiobankmeta.org/), for NICM (https://cvd.hugeamp.org/), for AF, for stroke (subtypes) from GIGASTROKE (https://www.ebi.ac.uk/gwas/publications/36180795), venous thromboembolism and abdominal aortic aneurysm from (https://www.globalbiobankmeta.org/), blood pressure from Evangelou et.al. (https://www.nature.com/articles/s41588-018-0205-x), glycemic traits, and lung function measurement were sourced from (http://www.nealelab.is/uk-biobank); type 2 diabetes from DIAGRAM; BMI from GIANT; CRP from (https://www.ebi.ac.uk/gwas/publications/30388399); the CKDGen consortium provided GWAS associations on estimated glomerular filtration rate, and chronic kidney disease (http://ckdgen.imbi.uni-freiburg.de/); Alzheimer’s disease data were sourced from Jansen et.al. (https://ctg.cncr.nl/software/summary_statistics), and drug prescriptions from (https://www.ebi.ac.uk/gwas/publications/31015401) Finally, the aggregate GWAS results for DCM can be requested from the GWAS corresponding authors.

## Code availability

Analyses were conducted using Python v3.7.10 (for GNU Linux), Pandas v1.3.5, Numpy v1.20.3, and Matplotlib 3.3.2. Scripts and data necessary to generate the illustrations have been deposited: 10.5522/04/28639193^38^.
