# Supplementary material for: A Mendelian randomization analysis of cardiac MRI measurements as surrogate outcomes for heart failure and atrial fibrillation
Source: Commun Med (Lond). 2025 Apr 19;5:130. doi: 10.1038/s43856-025-00855-1 (PMC12009341; doi:10.1038/s43856-025-00855-1)
Supplement: Supplementary file 1 — Supplementary Information [file 43856_2025_855_MOESM1_ESM.pdf]

A Mendelian randomization analysis of cardiac MRI measurements as surrogate outcomes for heart failure and atrial fibrillation

A F Schmidt *et. al.*

Contents

Supplementary Methods **2**

Supplementary Figures **4**

Supplementary Tables **8**

Supplementary References **11**

Supplementary Methods

Cardiac MRI GWAS

We sourced aggregate genetic data (i.e., point estimates and standard errors) from two UK biobank-based CMR studies: Ahlberg *et al.*[1], and Schmidt *et al.*[2].

- Ahlberg *et al.* leveraged a pre-trained convolutional neural network to derive five LA CMR traits in 35,658 UK biobank participants. People with established coronary heart disease or cardiomyopathy were excluded, as were people with a body mass index below 16 or above 40 kg/m^2. The median age of included participants was 64 (quartile 1, Q1: 58, Q3: 70), 48% were men, with a mean weight of 76 kg (standard deviation, SD: 10), and systolic/diastolic blood pressure of 130/77 mmHg (SD: 18/10). To prevent potential population stratification bias, people of non-European ancestry were excluded.
- Schmidt *et al.* leveraged a pre-trained convolutional neural network to derive 16 CMR traits in 36,548 UK biobank participants. People with established coronary heart disease, heart failure, or congenital heart disease were excluded. The average age of participants was 63.7 (SD: 7.6) years; the sample included 17,093 male subjects, with a mean weight of 76.0 kg (SD: 15.1), and systolic/diastolic blood pressure of 138.2/76.8 mmHg (SD: 18.4/10.0). Potential population stratification was accounted for through the application of a mixed-effects estimation method (BOLT-LMM) to address the inclusion of the 3.2% non-European ancestry participants.

Cardiac outcome GWAS

Aggregate genetic data (i.e., point estimates and standard errors) were sourced from genome-wide association studies on atrial fibrillation (AF), heart failure (HF), dilated cardiomyopathy (DCM), and non-ischemic cardiomyopathy (NICM). Below, we summarize the study-specific characteristics.

Atrial Fibrillation

Genetic associations with AF were sourced from Nielsen *et al.*[3], which enrolled 1,030,836 European participants, including 60,620 people with AF. The GWAS was based on a meta-analysis of five prospectively designed studies, as well as including a previous GWAS from Christophersen *et al.* 2017. The meta-analysis employed a bespoke study-specific definition of AF.

- The HUNT study defined AF based on the ICD-10/9 codes I48 and 427.3.
- The Michigan Genomics Initiative defined AF based on the ICD-9 code 427.31.
- The DiscovEHR Collaboration Cohort defined AF based on the ICD-10 code I48.
- The UK biobank defined AF based on the ICD-10/9 codes I48 and 427.3.
- The Christophersen *et al.* 2017 GWAS defined AF based on a combination of EHR diagnosis (e.g., using ICD codes) and ECG/Holter measurements.

Please see the following link for additional details.

Heart Failure

Genetic associations with HF were sourced from Wu *et al.*[4], part of the Global Biobank Meta-analysis Initiative (GBMI), which enrolled 1,014,441 European participants, of whom 52,496 developed HF. GBMI applied a uniform HF case definition using phecodes 428.2 (any heart failure), 428.3 (HFrEF), and 428.4 (HFpEF). The European ancestry GWAS was based on a meta-analysis of 10 prospectively designed biobanks: BioMe, BioVU, Estonian Biobank, FinnGen, HUNT, Lifelines, Michigan Genomics Initiative, Partners Biobank, UCLA Precision Health Biobank, and UK biobank. Please see the following link for additional details.

Dilated Cardiomyopathy

Genetic associations with DCM were sourced from the Garnier *et al.*[5] study, which enrolled 7,159 subjects, including 2,719 people with DCM. The meta-analysis of 9 studies, which excluded the UK biobank, defined DCM based on the presence of reduced ejection fraction and an enlarged left ventricular end-diastolic volume/diameter in the absence of significant coronary artery disease, intrinsic valvular disease, documented myocarditis, systemic disease (such as sarcoidosis), sustained arterial hypertension, or congenital malformation. Please see the following link for additional details.

Non-Ischemic Cardiomyopathy

Genetic associations with NICM were sourced from the Aragam *et al.*[6] study, which enrolled 395,972 subjects, of whom 1,816 developed NICM. This GWAS was exclusively conducted in the UK biobank, with NICM cases defined as an HF diagnosis with LV dysfunction in the absence of CAD. Here, LV dysfunction was defined as a diagnosis of DCM or LV failure. CAD was defined as a diagnosis of myocardial infarction or having received a coronary revascularisation. Please see the following link for additional details.

Mendelian randomisation on the association between heart failure liability on drug prescriptions

To explore potential mechanisms underlying the observed association between increased HF liability and type 2 diabetes, we conducted a focused Mendelian randomisation analysis. This analysis aimed to determine whether HF liability is linked to an increased likelihood of prescribing drugs known to lead to de novo type 2 diabetes, such as HMGCR inhibitors (i.e., statins)[7]. For this expanded the MR analysis genetic instruments were selected based on their association with HF, applying the same cut-offs and filters as described in the main manuscript. The GWAS by Wu *et al*. 2019 [8], which determined the genetic associations with drug prescriptions grouped by ATC code, was used as outcome source.

Supplementary Figures

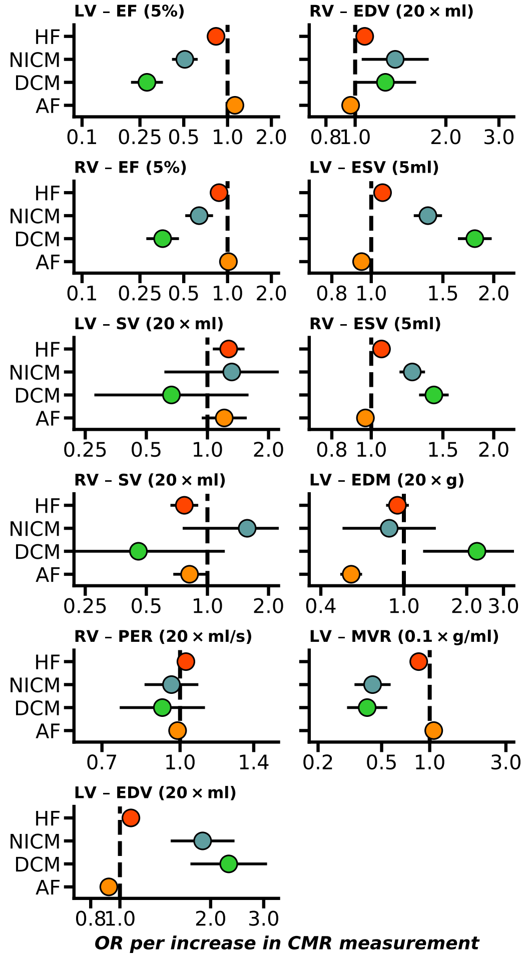

Supplementary Figure 1: Mendelian randomization Rucker-based estimates of biventricular CMR associations with the onset of HF and AF. Point estimates reflect odds ratios (OR) with 95% confidence intervals presented as horizontal line segments. LV: left-ventricle, RV: right-ventricle, EF: ejection fraction, SV: stroke volume, PFR: peak filling rate, PER: peak ejection rate, EDV/ESV: end-diastolic or end-systolic volumes, EDM: end-diastolic mass, MVR: mass-to-volume ratio. Outcome data were available for HF (heart failure, 52,496 cases), DCM (dilated cardiomyopathy, 2,719 cases), NICM (non-ischemic cardiomyopathy, 1,816 cases), and AF (atrial fibrillation, 60,620 cases).

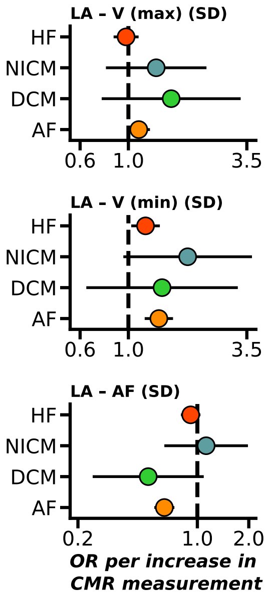

Supplementary Figure 2: Mendelian randomization Rucker-based estimates of atrial CMR associations with the onset of HF and AF. Point estimates reflect odds ratios (OR) with 95% confidence intervals presented as horizontal line segments. LA: left-atrial, V (max): maximum volume, V (min): minimum volume, AF: active emptying fraction. Outcome data were available for HF (heart failure, 52,496 cases), DCM (dilated cardiomyopathy, 2,719 cases), NICM (non-ischemic cardiomyopathy, 1,816 cases), and AF (atrial fibrillation, 60,620 cases).

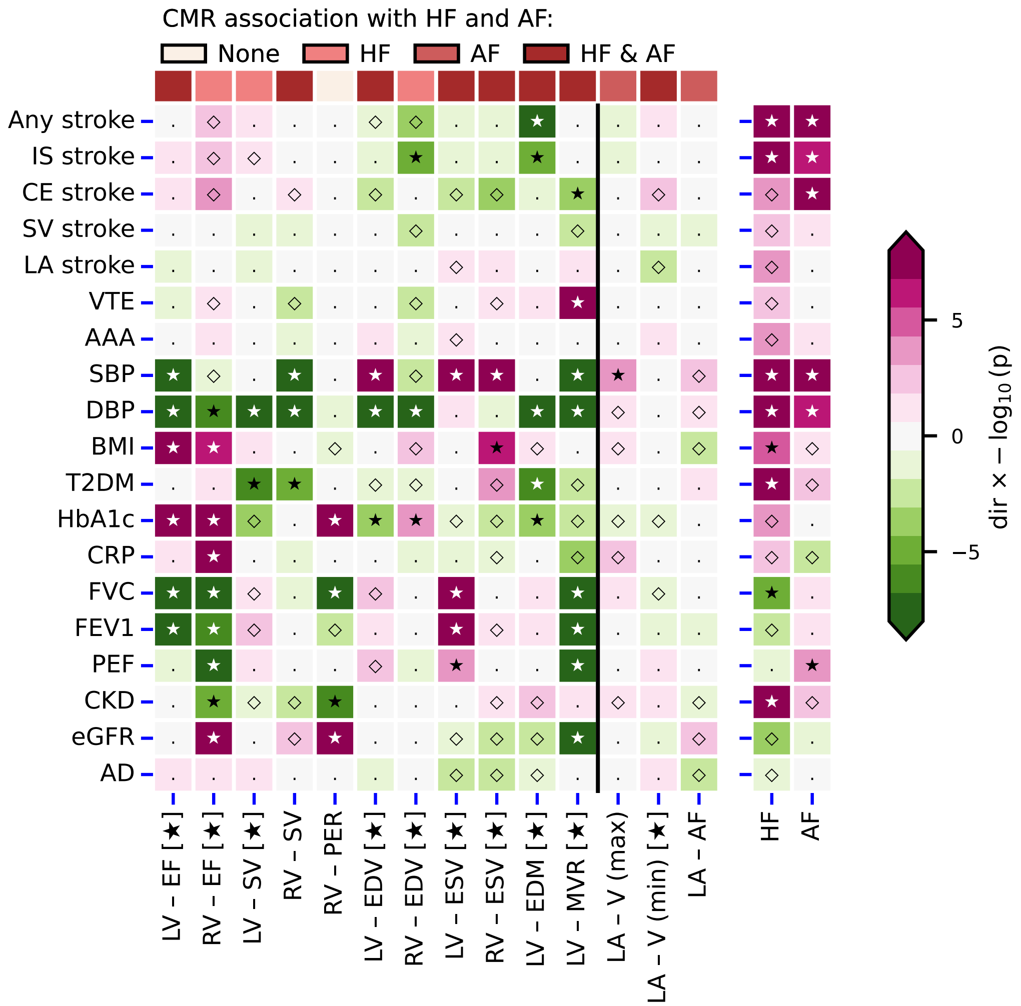

Supplementary Figure 3: A targeted Mendelian randomization Rucker-based phenome-wide scan contrasting the effects of changes in cardiac function and structure with effects of increased liability for HF or AF. P-values passing a 0.05 threshold are indicated by an open diamond, with stars indicating results passing a multiplicity corrected threshold. Cells are coloured by effect direction times $-\log_{10}$(p-value), with values truncated at ± 8 for display purposes. CMR measurements which a convincing cardiac association (based on a Kolmogorov-Smirnov) are indicated by a star appended to their x-axis label. The following abbreviations were used, LV: left-ventricle, RV: right-ventricle, LA: left-atrial, EF: ejection fraction, SV: stroke volume, PFR: peak filling rate, PER: peak ejection rate, EDV/ESV: end-diastolic or end-systolic volumes, EDM: end-diastolic mass, MVR: mass-to-volume ratio, V (max): maximum volume, V (min): minimum volume, TF: total emptying fraction, AF: active emptying fraction, PF: passive emptying fraction, AF: atrial fibrillation, HF: heart failure, IS: ischemic, CE: cardioembolic, SV: small vessel stroke, LA: larger artery, T2DM: type 2 diabetes, CKD: chronic kidney disease, VTE: venous thromboembolism, AAA: abdominal aortic aneurysm, SBP/DBP: systolic/diastolic blood pressure, BMI: body mass index, CRP: c-reactive protein, FVC: forced vital capacity, FEV1: forced expiratory volume, PEF: peak expiratory flow, eGFR: estimated glomerular filtration rate, HbA1c: glycated haemoglobin, AD: Alzheimer's dementia.

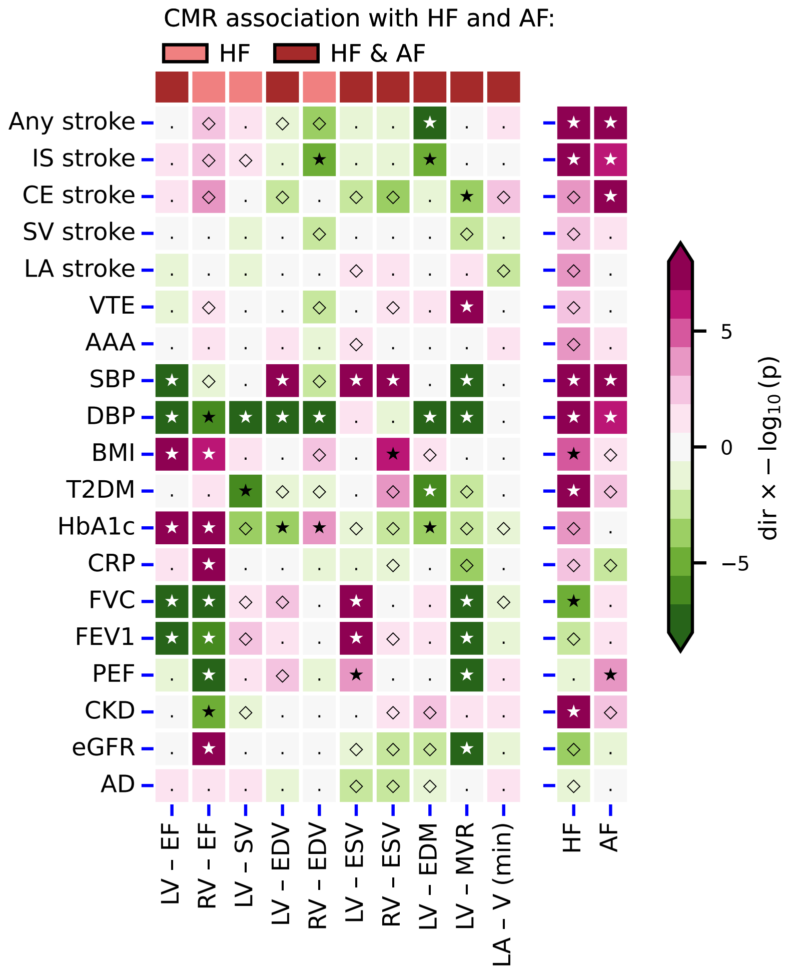

Supplementary Figure 4: A targeted Mendelian randomization Rucker-based phenome-wide scan contrasting the effects of changes in cardiac function and structure with effects of increased liability for HF or AF. P-values passing the 0.05 threshold are indicated by an open diamond, with stars indicating results passing a threshold. Cells are coloured by effect direction times $-\log_{10}$(p-value), with values truncated at ± 8 for display purposes. CMR measurements which a convincing cardiac association (based on a Kolmogorov-Smirnov) are indicated by a star appended to their x-axis label. The following abbreviations were used: LV: left ventricle, RV: right ventricle, LA: left-atrial, EF: ejection fraction, SV: stroke volume, PFR: peak filling rate, PER: peak ejection rate, EDV/ESV: end-diastolic or end-systolic volumes, EDM: end-diastolic mass, MVR: mass-to-volume ratio, V (max): maximum volume, V (min): minimum volume, TF: total emptying fraction, AF: active emptying fraction, PF: passive emptying fraction, AF: atrial fibrillation, T2DM: type 2 diabetes, CKD: chronic kidney disease, VTE: venous thromboembolism, AAA: abdominal aortic aneurysm, SBP/DBP: systolic/diastolic blood pressure, BMI: body mass index, CRP: c-reactive protein, FVC: forced vital capacity, FEV1: forced expiratory volume, PEF: peak expiratory flow, eGFR: estimated glomerular filtration rate, HbA1c: glycated haemoglobin, AD: Alzheimer's dementia.

Supplementary Tables

Supplementary Table 1: Mendelian randomization median estimates of the association between heart failure liability and drug prescription, including cardiovascular, and non-cardiovascular negative control prescriptions.

Drug prescription	OR (95%CI	P-value
N02BE: Anilides	0.95 (0.87; 1.04)	2.9×10^{-1}
N06A: Antidepressants	0.98 (0.87; 1.09)	6.8×10^{-1}
L04: Immunosuppressants	1.17 (0.87; 1.59)	3.0×10^{-1}
C09: Agents Acting On The Renin Angiotensin System	1.54 (1.36; 1.75)	1.5×10^{-11}
C02: Antihypertensives	1.43 (1.12; 1.84)	4.6×10^{-3}
C07: Beta Blocking Agents	1.54 (1.32; 1.79)	3.1×10^{-8}
C08: Calcium Channel Blockers	1.25 (1.08; 1.45)	2.6×10^{-3}
C03: Diuretics	1.18 (1.04; 1.35)	1.3×10^{-2}
C10AA: HMG Coa Reductase Inhibitors	1.28 (1.17; 1.40)	3.5×10^{-8}

General:
The presciption GWAS data were sourced from Wu *et al.* 2019 [8], analysing prescriptions by the indicated ATC code.

Supplementary Table 2: An overview of the outcome GWAS and whether these sourced data from the UK biobank (UKB).

Fist Author	Trait	Studies	Used UKB	UKB percentage of total sample	UKB percentage of total cases
Wu et al.	HF	BioMe, BioVU, Estonian Biobank, FinnGen, HUNT, Lifelines, Michigan Genomics Initiative, Partners biobank, UCLA Precision Health Biobank, and UK Biobank	Yes	NA	NA
Nielsen et al.	AF	HUNT, Michigan Genomics Initiative, DiscovEHR Collaboration Cohort, UK Biobank, Christophersen et al. 2017	Yes	67.3	24.45
Garnier et al.	DCM	CARDIGENE, EUROGENE, PHRC, Berlin Cohort, German EUROGENE, Royal Brompton and Harefield Hospitals NHS Foundation Trust, Pavia, MAGNet, PPS3, KORA	No	0.0	0.00
Aragram et al. 2019	NICM	UKB	Yes	100.0	100.00
Mishra et al.	Any stroke	Over 45	Yes	22.9	2.66
Mishra et al.	SV stroke	Over 45	No	0.0	0.00
Mishra et al.	CE stroke	Over 45	No	0.0	0.00
Mishra et al.	LA stroke	Over 45	No	0.0	0.00
Mishra et al.	IS stroke	Over 45	Yes	23.2	2.55
Wolford et al.	VTE	BioMe, BioVU, ETBB, FinnGen, MGI, UCLA, UKB	Yes	NA	NA
GBMI	AAA	BioMe, BioVU, CCPM, DECODE, ESTBB, FinnGen, HUNT, MGB, MGI, UKB	Yes	NA	NA
Evangelou et al.	SBP/DBP	UKB, ICBP	Yes	60.5	0.00
Pulit et al.	BMI	GIANT, UKB	Yes	60.1	0.00
Mahajan et al.	T2DM	32 studies	Yes	47.2	25.79
Neale	HbA1c	UKB	Yes	100.0	0.00
Ligthart et al.	CRP	72 studies	No	0.0	0.00
Neale	Lung functions	UKB	Yes	100.0	0.00
Wuttke et al.	CKD	Over 80 studies	No	0.0	0.00
Wuttke et al.	eGFR	Over 80 studies	No	0.0	0.00
Kunkle et al.	AD	46 studies	No	0.0	0.00

General:
NA (not available) was used when we could not determine the number of UKB participants which contributed to the GWAS meta-analysis.

Supplementary References

[1] Gustav Ahlberg *et al*. "Genome-wide association study identifies 18 novel loci associated with left atrial volume and function." In *European Heart Journal* 42.44 (Aug. 2021), pp. 4523–4534. ISSN: 1522-9645. DOI: 10.1093/eurheartj/ehab466.

[2] Amand F. Schmidt *et al*. "Druggable proteins influencing cardiac structure and function: Implications for heart failure therapies and cancer cardiotoxicity." In *Science Advances* 9.17 (Apr. 2023). ISSN: 2375-2548. DOI: 10.1126/sciadv.add4984.

[3] Jonas B. Nielsen *et al*. "Biobank-driven genomic discovery yields new insight into atrial fibrillation biology." In *Nature genetics* 50 (9 Sept. 2018), pp. 1234–1239. ISSN: 1546-1718. DOI: 10.1038/s41588-018-0171-3. ppublish.

[4] Kuan-Han H. Wu *et al*. "Polygenic risk score from a multi-ancestry GWAS uncovers susceptibility of heart failure." In (Dec. 2021). DOI: 10.1101/2021.12.06.21267389.

[5] Sophie Garnier *et al*. "Genome-wide association analysis in dilated cardiomyopathy reveals two new players in systolic heart failure on chromosomes 3p25.1 and 22q11.23." In *European Heart Journal* 42.20 (Mar. 2021), pp. 2000–2011. ISSN: 1522-9645. DOI: 10.1093/eurheartj/ehab030.

[6] Krishna G. Aragam *et al*. "Phenotypic Refinement of Heart Failure in a National Biobank Facilitates Genetic Discovery." In *Circulation* 139.4 (Jan. 2019), pp. 489–501. ISSN: 1524-4539. DOI: 10.1161/circulationaha.118.035774.

[7] Daniel I Swerdlow *et al*. "HMG-coenzyme A reductase inhibition, type 2 diabetes, and bodyweight: evidence from genetic analysis and randomised trials." In *The Lancet* 385.9965 (Jan. 2015), pp. 351–361. ISSN: 0140-6736. DOI: 10.1016/s0140-6736(14)61183-1.

[8] Yeda Wu *et al*. "Genome-wide association study of medication-use and associated disease in the UK Biobank." In *Nature Communications* 10.1 (Apr. 2019). ISSN: 2041-1723. DOI: 10.1038/s41467-019-09572-5.
