# Supplementary material for: A Mendelian randomization analysis of cardiac MRI measurements as surrogate outcomes for heart failure and atrial fibrillation
Source: Commun Med (Lond). 2025 Apr 19;5:130. doi: 10.1038/s43856-025-00855-1 (PMC12009341; doi:10.1038/s43856-025-00855-1)
Supplement: Supplementary file 2 — Description of Additional Supplementary Files [file 43856_2025_855_MOESM2_ESM.pdf]

Description of Additional Supplementary Files

File name: Data 1
Description: Mendelian randomization estimates of the effects CMR-measured changes in cardiac function and structure have on the development of cardiac diseases.

File name: Data 2
Description: Mendelian randomization median estimates of the effects CMR measured changes in cardiac function and structure have on the development of cardiac diseases.

File name: Data 3
Description: Mendelian randomization estimates of the effects CMR measured changes in cardiac function and structure have on the development of non-cardiac diseases or traits.

File name: Data 4
Description: Mendelian randomization median estimates of the effects CMR measured changes in cardiac function and structure have on the development of non-cardiac diseases or traits.

File name: Data 5
Description: Mendelian randomization estimates of the effects an increase in liability of atrial fibrillation or heart failure has on the development of non-cardiac diseases or traits.

File name: Data 6
Description: Mendelian randomization median estimates of the effects an increase in liability of atrial fibrillation or heart failure has on the development of non-cardiac diseases or traits.

File name: Data 7
Description: The genetic variants used for in the Mendelian randomization analyses.
