# Supplementary material for: A Mendelian randomization analysis of cardiac MRI measurements as surrogate outcomes for heart failure and atrial fibrillation
Source: Commun Med (Lond). 2025 Apr 19;5:130. doi: 10.1038/s43856-025-00855-1 (PMC12009341; doi:10.1038/s43856-025-00855-1)
Supplement: Supplementary file 10 — REPORTING SUMMARY [file 43856_2025_855_MOESM10_ESM.pdf]

nature portfolio

Corresponding author(s): A F Schmidt

Last updated by author(s): Mar 21, 2025

Reporting Summary

Nature Portfolio wishes to improve the reproducibility of the work that we publish. This form provides structure for consistency and transparency in reporting. For further information on Nature Portfolio policies, see our Editorial Policies and the Editorial Policy Checklist.

Statistics

For all statistical analyses, confirm that the following items are present in the figure legend, table legend, main text, or Methods section.

n/a	Confirmed	
☐	☒	The exact sample size (*n*) for each experimental group/condition, given as a discrete number and unit of measurement
☐	☒	A statement on whether measurements were taken from distinct samples or whether the same sample was measured repeatedly
☐	☒	The statistical test(s) used AND whether they are one- or two-sided<br>*Only common tests should be described solely by name; describe more complex techniques in the Methods section.*
☒	☐	A description of all covariates tested
☐	☒	A description of any assumptions or corrections, such as tests of normality and adjustment for multiple comparisons
☐	☒	A full description of the statistical parameters including central tendency (e.g. means) or other basic estimates (e.g. regression coefficient) AND variation (e.g. standard deviation) or associated estimates of uncertainty (e.g. confidence intervals)
☐	☒	For null hypothesis testing, the test statistic (e.g. *F*, *t*, *r*) with confidence intervals, effect sizes, degrees of freedom and *P* value noted<br>*Give P values as exact values whenever suitable.*
☒	☐	For Bayesian analysis, information on the choice of priors and Markov chain Monte Carlo settings
☒	☐	For hierarchical and complex designs, identification of the appropriate level for tests and full reporting of outcomes
☐	☒	Estimates of effect sizes (e.g. Cohen's *d*, Pearson's *r*), indicating how they were calculated

Our web collection on statistics for biologists contains articles on many of the points above.

Software and code

Policy information about availability of computer code

Data collection	Data were collected from publicly available GWAS repositories; no specialist software was used for this.
Data analysis	Analyses were conducted using Python

For manuscripts utilizing custom algorithms or software that are central to the research but not yet described in published literature, software must be made available to editors and reviewers. We strongly encourage code deposition in a community repository (e.g. GitHub). See the Nature Portfolio guidelines for submitting code & software for further information.

Data

Policy information about availability of data

All manuscripts must include a data availability statement. This statement should provide the following information, where applicable:

- Accession codes, unique identifiers, or web links for publicly available datasets
- A description of any restrictions on data availability
- For clinical datasets or third party data, please ensure that the statement adheres to our policy

Genetic data for the phewas of non-cardiac traits was sourced for stroke (subtypes) from MEGASTROKE (http://www.megastroke.org/index.html), venous thromboembolism and abdominal aortic aneurysm from (https://www.globalbiobankmeta.org/), blood pressure from Evangelou et.al. (https://www.nature.com/articles/s41588-018-0205-x), glycemic traits, and lung function measurement were sourced from (http://www.nealelab.is/uk-biobank); type 2 diabetes from DIAGRAM (http://diagram-consortium.org/index.html); BMI from GAINT (https://portals.broadinstitute.org/collaboration/giant/index.php/

GIANT_consortium_data_files); CRP from (https://www.ebi.ac.uk/gwas/publications/30388399); the CKDGen consortium provided GWAS associations on estimated glomerular filtration rate (eGFR), and chronic kidney disease (http://ckdgen.imbi.uni-freiburg.de/); Alzheimer's disease data was sourced from Jansen et.al. (https://ctg.cncr.nl/software/summary_statistics) and Kunkle et.al. (https://www.nature.com/articles/s41588-019-0358-2); Lewy body dementia from (https://www.ebi.ac.uk/gwas/publications/33589841);

Human research participants

Policy information about studies involving human research participants and Sex and Gender in Research.

Reporting on sex and gender	We analyzed aggregated genetic data which were not available stratified for men and women.
Population characteristics	We analyzed aggregated genetic data based on participants from European ancestry
Recruitment	We analyzed aggregated genetic data
Ethics oversight	The individual studies reanalysed by us all obtained relevant ethical approval

Note that full information on the approval of the study protocol must also be provided in the manuscript.

Field-specific reporting

Please select the one below that is the best fit for your research. If you are not sure, read the appropriate sections before making your selection.

☒ Life sciences ☐ Behavioural & social sciences ☐ Ecological, evolutionary & environmental sciences

For a reference copy of the document with all sections, see nature.com/documents/nr-reporting-summary-flat.pdf

Life sciences study design

All studies must disclose on these points even when the disclosure is negative.

Sample size	We did not perform any sample size calculations.
Data exclusions	To improve computational stability, low MAF (below 0.01) variants were excluded (apriori defined).
Replication	We did not replicate our findings
Randomization	While genes cannot be randomly allocated, they are inherited in a random fashion (following Mendel's laws of inheritance).
Blinding	While subjects cannot be actively blinded from their genotype, most subjects would have been unaware of their genotype. Should they have been aware, the implication (if any) on disease would in most cases be insufficiently clear to result in any marked bias.

Reporting for specific materials, systems and methods

We require information from authors about some types of materials, experimental systems and methods used in many studies. Here, indicate whether each material, system or method listed is relevant to your study. If you are not sure if a list item applies to your research, read the appropriate section before selecting a response.

Materials & experimental systems

n/a	Involved in the study	
☒	☐	Antibodies
☒	☐	Eukaryotic cell lines
☒	☐	Palaeontology and archaeology
☒	☐	Animals and other organisms
☒	☐	Clinical data
☒	☐	Dual use research of concern

Methods

n/a	Involved in the study	
☒	☐	ChIP-seq
☒	☐	Flow cytometry
☒	☐	MRI-based neuroimaging
